# Supplementary material for: Cooperative binding mitigates the high-dose hook effect
Source: BMC Syst Biol. 2017 Aug 14;11:74. doi: 10.1186/s12918-017-0447-8 (PMC5556679; doi:10.1186/s12918-017-0447-8)
Supplement: Supplementary file 1 — Supplemental text. (PDF 106 kb) [file 12918_2017_447_MOESM1_ESM.pdf]

Supplemental Information: Cooperative Binding Mitigates the High-Dose Hook Effect

R. Dutta Roy, C. Rosenmund, M.I. Stefan

1 Exploration of parameter space

In order to explore how the high-dose Hook effect is dependent on the parameters used in our model, we repeated the simulations done for the figure entitled "Reduced hook effect in cooperative (wt) calmodulin" with a range of parameter values. First, we varied all dissociation constants by factors of 10. The results (see figure 1) show that a high-dose Hook effect is observed in all the cases. Since the peak of CaM-Ca4 concentrations is highest at low K_d values (with tight binding), the decrease at increasing calmodulin concentrations is more severe in absolute terms.

Next, we examined the impact of the allosteric equilibrium constant, L. High L values mean that the ratio of calmodulin molecules in the absence of Calcium is more highly skewed towards the T state (rather than the R state). $L = 1$ means that the T state and R state are equally populated in the absence of ligand. As seen in figure 2, at lower L values the R state is favoured, resulting in a higher overall peak (due to the higher affinity of the R state), but also a strong high-dose hook effect (because cooperativity between the T and R state is what mitigates the high-dose hook effect.)

2 Fractional occupancy and fraction of protein in the R state

The high-dose hook effect is a phenomenon relating to full saturation of a multivalent molecule. In the paper, we show how the high-dose hook effect is stronger for calmodulin in the presence of a high concentration of CaMKII (140 μM) and less strong for calmodulin in the presence of a lower concentration (1 μM) of CaMKII or in the absence of CaMKII. Here, we explore whether this difference is also evident in total fractional occupancy (i.e. the average number of occupied binding sites per molecule of calmodulin) or in the fraction of calmodulin that is present in the R state. Figure 3 shows fractional occupancy at equilibrium as a function of initial calmodulin concentration. As expected, it declines monotonically, and it is a bit higher in the presence of CaMKII, where the high-affinity states are favoured. Figure 4 shows fraction of calmodulin in the R state at

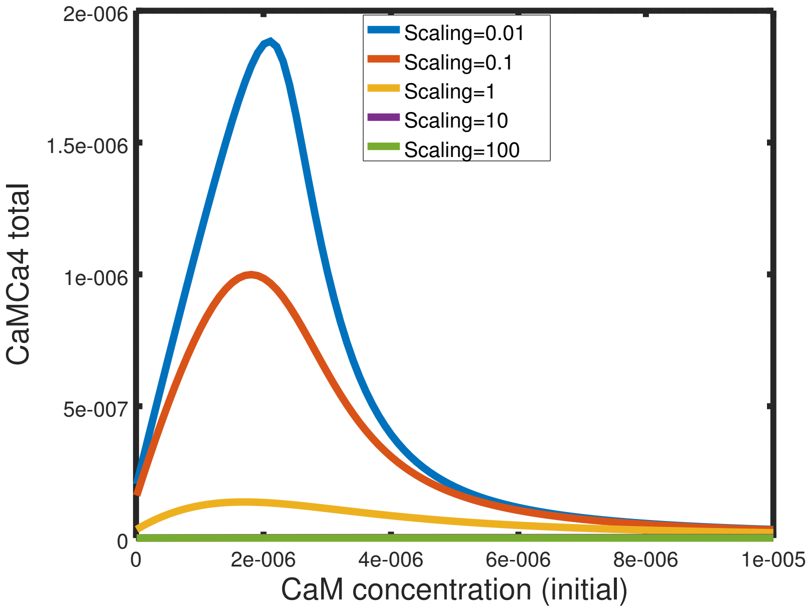

Figure 1: All K_d values were multiplied with a scaling factor. Scaling factor 1 corresponds to the figure as shown in the paper. Lower scaling factors indicate tighter binding.

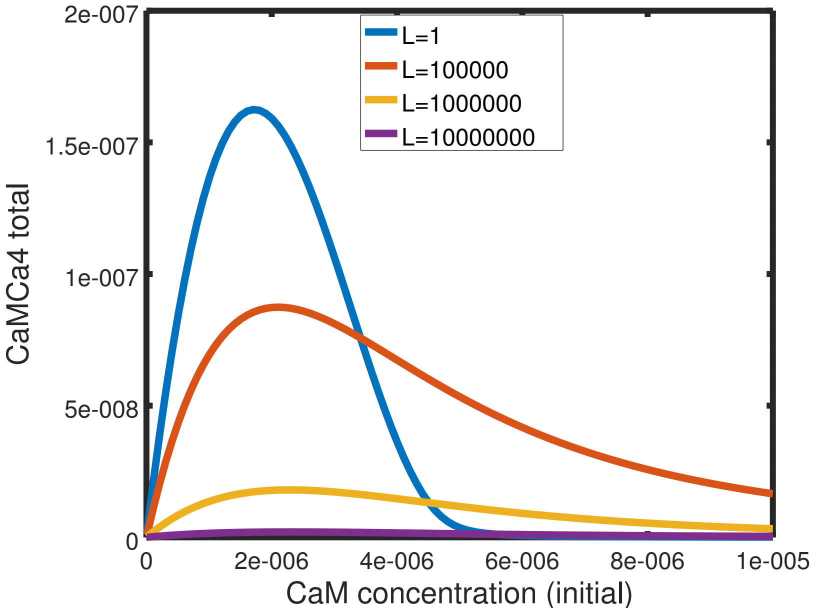

Figure 2: L value used in the paper was 20670. High L values mean that the T state is more strongly favoured over the R state in the absence of ligand. $L = 1$ means that there are equal amounts of R and T in the *apo* state.

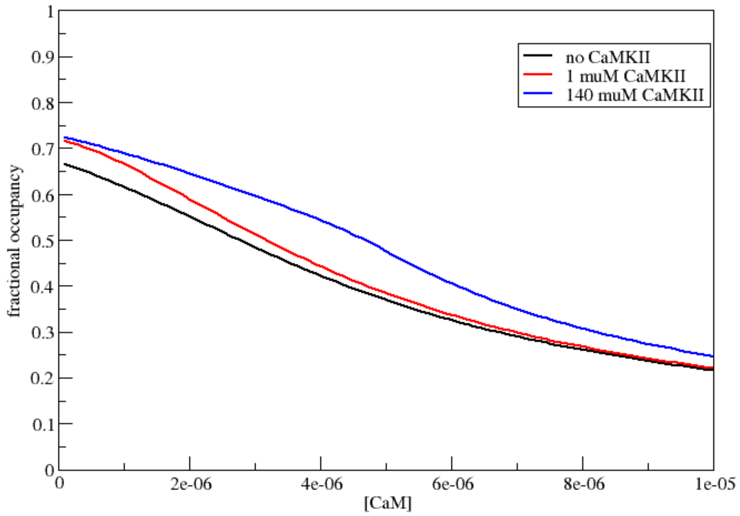

Figure 3: $\bar{Y}$ (fraction of occupied binding sites) as a function of calmodulin concentration at different concentrations of CaMKII.

equilibrium as a function of initial calmodulin concentration. In the presence of large amounts of CaMKII, the R state prevails even at higher calmodulin concentrations. In the absence of CaMKII or in the presence of low concentrations of CaMKII, R and T states both exist, thereby rendering calmodulin cooperative and thereby mitigating the high-dose hook effect (see main text).

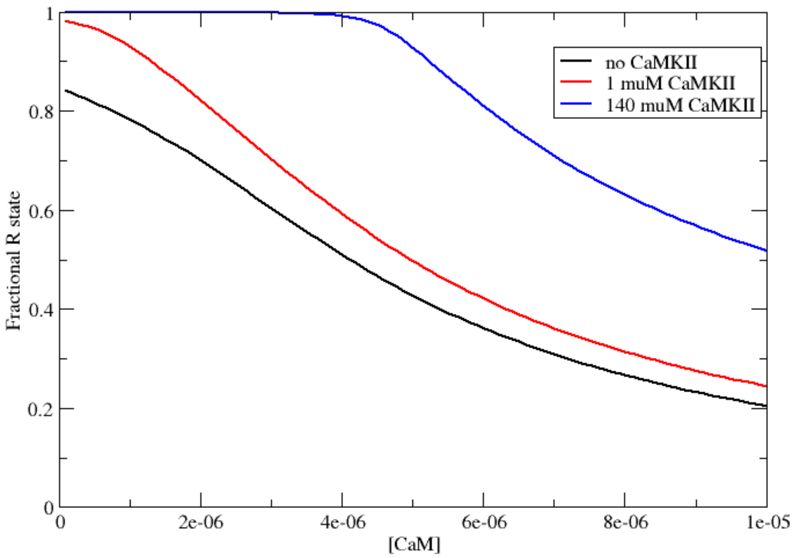

Figure 4: $\bar{R}$ (fraction of calmodulin in the R state) as a function of calmodulin concentration at different concentrations of CaMKII.
